# Evaluation of glymphatic system activity by diffusion tensor image analysis along the perivascular space in presbycusis

**DOI:** 10.1111/cns.14458

**Published:** 2023-09-08

**Authors:** Kaixi Xu, Juan Zhang, Chunhua Xing, Xiaomin Xu, Xindao Yin, Yuanqing Wu, Xinjian Chen, Yu‐Chen Chen

**Affiliations:** ^1^ Department of Radiology Lianyungang Traditional Chinese Medicine Hospital Affiliated to Nanjing University of Chinese Medicine Lianyungang China; ^2^ Department of Neurology, Nanjing Yuhua Hospital Yuhua Branch of Nanjing First Hospital Nanjing China; ^3^ Department of Radiology, Nanjing First Hospital Nanjing Medical University Nanjing China; ^4^ Department of Otolaryngology, Nanjing First Hospital Nanjing Medical University Nanjing China

**Keywords:** cognitive decline, diffusion tensor imaging, glymphatic system, perivascular space, presbycusis

## Abstract

**Purpose:**

Previous studies have suggested that presbycusis (age‐related hearing loss) is accompanied with cognitive decline and dementia. However, the neural mechanism underlying the cognitive decline in presbycusis remains unclear. This study aimed to evaluate the glymphatic system function in presbycusis patients compared to healthy controls using diffusion tensor imaging (DTI) with the perivascular space (DTI‐ALPS) method.

**Methods:**

DTI scans were obtained from 30 presbycusis patients with cognitive decline (PCD), 30 presbycusis patients with no cognitive decline (PNCD) and 40 age‐, gender‐, and education‐matched healthy controls (HCs). The DTI‐ALPS index was calculated for each group. We evaluated the differences in the DTI‐ALPS index among PCD, PNCD and HCs. In addition, we conducted a correlation analysis between the DTI‐ALPS index and cognitive performance.

**Results:**

There were significant differences of the DTI‐ALPS index among three groups. Post‐hoc analysis suggested that the DTI‐ALPS index in PCD was significantly lower patients in relative to PNCD and HCs (1.49147 vs. 1.57441 vs. 1.62020, *p* < 0.001). After correcting for age, gender, and education, the DTI‐ALPS index is positively correlated with the MoCA scores (rho = 0.426, *p* = 0.026).

**Conclusion:**

Presbycusis patients with cognitive impairment exhibited decreased glymphatic activity than those without cognitive impairment and HCs. The DTI‐ALPS index may provide useful disease progression or treatment biomarkers for patients with presbycusis as an indicator of modulation of glymphatic activity.

## Graphical abstract

Presbycusis patients with cognitive impairment exhibited decreased glymphatic activity than those without cognitive impairment and healthy controls. The DTI‐ALPS index may provide useful disease progression or treatment biomarkers for patients with presbycusis as an indicator of modulation of glymphatic activity.

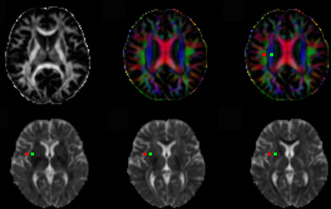

## 1 INTRODUCTION

Presbycusis (progressive bilateral high‐frequency sensorineural hearing loss) has become a major public issue frequently accompanied with social isolation, communication, language and speech processing problems that affect elderly adults.^1^, ^2^ The main clinical features of presbycusis are characterized by slow central processing of auditory information, impaired localization of sound sources, and reduced ability to distinguish speech in noisy environments.^3^ Previous studies have proved that presbycusis is independently associated with cognitive impairment and increasing risk of dementia.^4^, ^5^ However, the neural mechanism underlying cognitive decline in presbycusis still requires to be illustrated.

The glymphatic system is a recently discovered unique network of the central nervous system (CNS), which is pivotal for clearing the brain of protein waste products.^6^ In this system, cerebrospinal fluid (CSF) and interstitial fluid (ISF) interchange by influx of CSF along the loose fibrous matrix of perivascular spaces, facilitated by aquaporin‐4 (AQP4) water channels.^7^ Then, the pathway directs flow towards the venous perivascular and perineuronal spaces and ultimately enters the meningeal and cervical lymphatic drainage vessels. Furthermore, the glymphatic system is also involved in β‐amyloid (Aβ) clearance.^8^ When the metabolism of Aβ is out of line and the clearance function is damaged, the balance is broken, and the deposition and abnormal accumulation of Aβ cause a series of damages, which will result in cognitive impairment.^9^ Glymphatic system dysfunction has been recently implicated in various neurological diseases, such as Alzheimer's disease (AD), Parkinson's disease (PD) and stroke.^10^, ^11^, ^12^, ^13^, ^14^ However, no studies have investigated glymphatic system function in presbycusis to date.

The glymphatic system has been visualized using various imaging approaches,^6^, ^15^, ^16^ including brain section microscopy, transcranial mesoscopic imaging, proton emission tomography (PET) and gadolinium‐based contrast‐enhanced magnetic resonance imaging (MRI). Derived from MRI, diffusion tensor imaging (DTI) along the perivascular space (DTI‐ALPS) has been proposed as an index for assessing glymphatic system function.^17^ It evaluates the motion of water molecules in the direction of the perivascular space by measuring diffusivity using the diffusion tensor method. It relies on the fact that, at the level of the lateral ventricle body, the perivascular space along with the medullary veins lies orthogonal to the projection and association fibers. Glymphatic system dysfunction with histological changes will affect projection and association fibers.^18^ Previous studies have demonstrated the DTI‐ALPS index as a potential biomarker for glymphatic system function, with a lower value indicating reduced activity.^17^ The DTI‐ALPS is not invasive and does not require gadolinium‐based contrast administration, which has been recently applied in AD,^17^ PD,^19^ mild traumatic brain injury,^20^ and obstructive sleep apnea.^21^ Most studies focused on the relationship between the ALPS index and cognition. Nevertheless, no previous study investigated the relationship between glymphatic system function and cognitive impairment in presbycusis.

Therefore, this study aimed to employ the DTI‐ALPS index to assess glymphatic system function in presbycusis patients with or without cognitive decline and healthy controls (HCs). Here, we assumed that presbycusis patients might have glymphatic system dysfunction, which is associated with cognitive impairment.

## 2 MATERIALS AND METHODS

### 2.1 Subjects

All the subjects provided written informed consent before their participation in the study protocol, 100 participants (all right handed and educated for at least 8 years) were enrolled in this study, which included 60 presbycusis patients recruited from the department of otolaryngology and 40 age‐, gender‐, and education‐matched HCs recruited through community health census or online advertising. Hearing loss was assessed by the speech‐frequency pure tone average (PTA) of thresholds at the frequencies of 0.25, 0.5, 1, 2, 4, and 8 kHz in the better‐hearing ears. The PTA value of 25 dB was accepted as the normal hearing threshold limit. Inclusion criteria of the presbycusis were average PTA > 25 dB in the better hearing ear and age≧60 years. Tympanometry was performed with a Madsen Electronics Zodiac 901 Middle Ear Analyzer (GN Otometrics) to confirm normal middle‐ear function. Approval for the study was obtained from the Research Ethics Committee of Nanjing Medical University.

Exclusion criteria included the following: (1) ear diseases that affected hearing threshold, including tinnitus, hyperacusis^22^ and Meniere's disease^23^; (2) a history of ototoxic drug therapy, otologic surgery, noise exposure or hearing aid use; (3) conductive hearing loss (a mean air‐bone difference at 0.5, 1, 2, and 4 kHz) > 10 dB in one or both ears; and (4) severe smoking, alcohol abuse, brain damage, stroke, Alzheimer's disease, Parkinson's disease, major depression, epilepsy, mental or neurological disorders, major diseases (such as anemia, thyroid dysfunction, cancer); (5) MRI contraindications.

### 2.2 Neuropsychological assessment

A comprehensive test of cognitive status was performed on all participants using Montreal Cognitive Assessment (MoCA), including eight cognitive domains: visual space and executive function, attention, memory, naming, abstract thinking, language, delayed recall and orientation. The test was carried out in a quiet environment and all the subjects were expected to be relaxed and conscious. MoCA is commonly used to screen for Mild Cognitive Impairment (MCI), which has high sensitivity.^24^ The MoCA test result has a total score of 30 points, with a final score ≥ 26 being considered normal. According to the MoCA score, the presbycusis patients were divided into 30 presbycusis with cognitive decline (PCD) and 30 presbycusis with no cognitive decline (PNCD).

### 2.3 DTI data acquisition

Diffusion imaging was acquired using a 3.0‐Tesla MR imaging system (Magnetom Prisma, Siemens Healthcare) with a 64‐channel receiver array head coil. During scanning, the subjects were supposed to lie quietly with their eyes closed and avoid head movement during the scan, but not to fall asleep or think about anything special. To reduce head motion and scanner noise, foam pad and earplugs were used. According to the manufacturer's specifications, the earplugs (Hearos Ultimate Softness Series) could attenuate scanner noise by almost 32 dB. DTI images were obtained using a single‐shot echo planar imaging (EPI) sequence. The scan parameters were as follows: TR = 10s; TE = 95 ms; slices = 70; slice thickness = 2 mm; gap = 0; FA = 90°; *b*‐values = 0 and 1000 s/mm^2^; diffusion gradient directions = 30; matrix = 128 × 128; FOV = 256 mm × 256 mm. Structural images were obtained using a magnetization‐prepared rapid gradient echo (MP2RAGE) sequence and the following scan parameters: TR/ TE = 5000/2.98 ms, slices = 176, thickness = 1 mm, gap = 0 mm, FA = 90°, acquisition matrix = 256 × 256, and FOV = 256 mm × 256 mm.

### 2.4 DTI data preprocessing

Individual raw MRI data in the digital imaging and communications in medicine (DICOM) file format were retained. The DICOM files were converted to the NIFTI file format using the MRIcron program. Then, we preprocessed the DTI with a DSI studio program as follows^25^: (1) read the DICOM files and perform a quality inspection, (2) set up a mask to filter out the background region, increase the reconstruction efficacy, and facilitate further visualization, and (3) perform reconstruction with the DTI method to characterize the major diffusion direction of the fibers.

### 2.5 DTI‐ALPS index calculation

**FIGURE 1:**
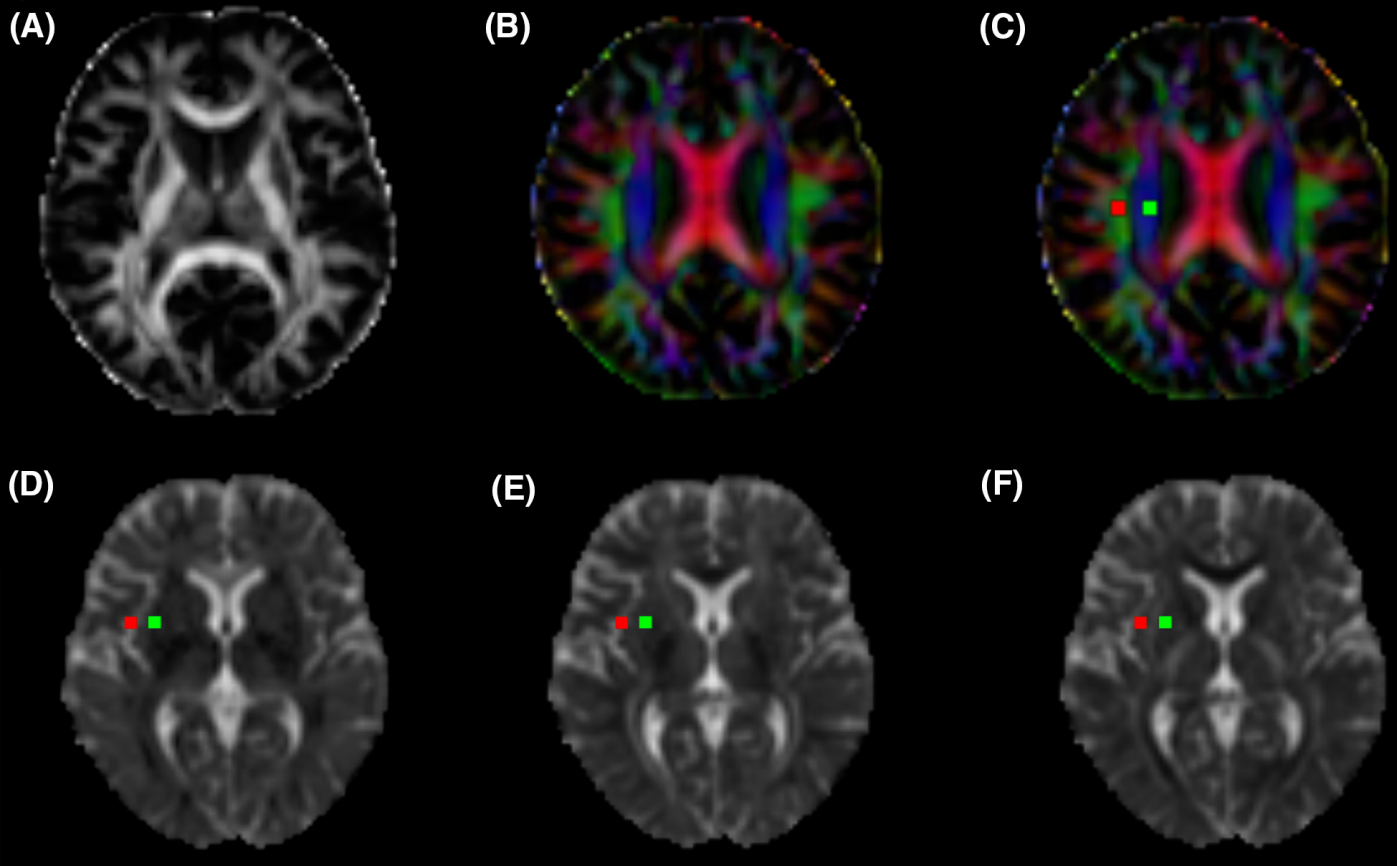
DTI‐ALPS calculation method. (A) FA original map; (B) FA color map; (B) Two 5‐mm‐diameter regions of interest were drawn at the areas of the projection, and association neural fibers in FA map. The ROIs in the FA maps were transferred to the Dx (D); Dy (E); and Dz (F) maps.

Figure 1 shows the process for obtaining the DTI‐ALPS index.^17^ We first drew a region of interest (ROI) in a rectangular shape. Then, we obtained the fiber orientation and diffusivities of the three directions along the x‐, y‐, and z‐axes as voxel levels at the ROI. Of the several voxels, we selected one ROI for each fiber on the same x‐axis (projection and association fibers), which showed the maximum orientation in each fiber. The DTI‐ALPS index was calculated using the following formula:

$$\text{ALPS index}=\frac{\text{mean}\;\left(D_{\text{xxproj}},D_{\text{xxassoc}}\right)}{\text{mean}\;\left(D_{\text{yyproj}},D_{\text{zzassoc}}\right)}$$

*D*_xxproj_: diffusivity along the x‐axis in the projection fiber.
*D*_xxassoc_: diffusivity along the x‐axis in the association fiber.
*D*_yyproj_: diffusivity along the y‐axis in the projection fiber.
*D*_zzassoc_: diffusivity along the z‐axis in the association fiber.

The average value of the bilateral ALPS index would be calculated as a measure of the glymphatic function of the basal ganglion area. A high ALPS index represents a good diffusivity along perivascular space, indicating a good glymphatic function.

### 2.6 Statistical analysis

Statistical analyses were performed using SPSS 25.0 (SPSS, Inc.). Categorical variables were investigated with a chi‐squared test. The normality of distribution was assessed using the Shapiro–Wilk test. Nonparametric tests were applied if the data were identified as not normally distributed, while normally distributed continuous variables were investigated with a one‐way analysis of variance test (ANOVA) for three groups. For each group, we calculated the DTI‐ALPS‐index and correlated them with the MoCA scores of the subjects, which was conducted with a two‐tailed Spearman's correlation with the effect of age, gender, and education adjusted. Interobserver reliability of manual DTI‐ALPS‐index was evaluated using intraclass correlation coefficient (ICC) with a two‐way random model. All data are presented as mean ± standard deviation (SD), and *p* < 0.05 was statistically significant.

## 3 RESULTS

**FIGURE 2:**
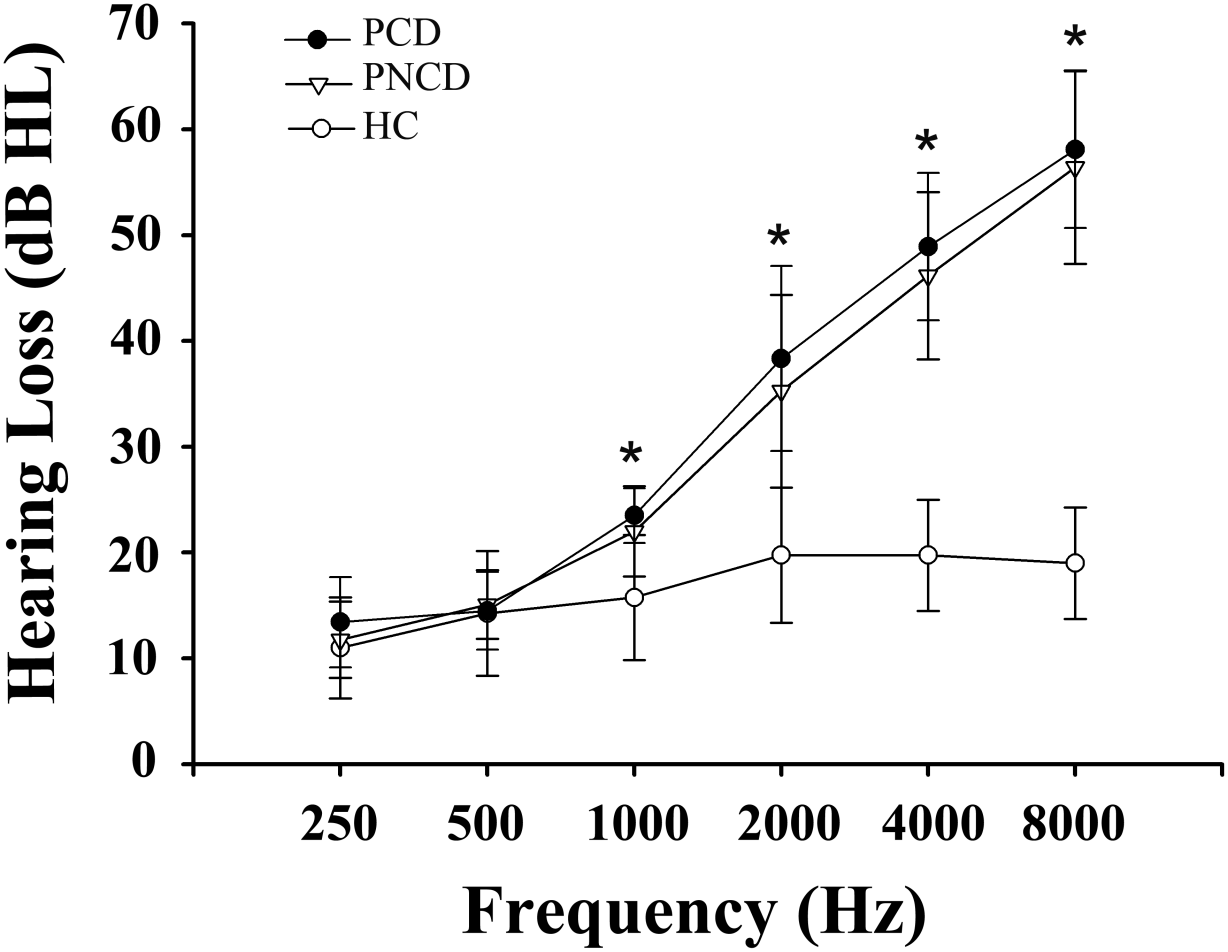
Average hearing thresholds of PCD, PNCD, and HC. The hearing thresholds were significantly higher in PCD and PNCD than HC (*p < 0.001; 500–8000 Hz). Data are presented as Mean ± SD. HC, healthy controls; PCD, presbycusis patients with cognitive decline; PNCD, presbycusis patients with no cognitive decline.

The demographics and clinical characteristics of three groups were summarized in Table 1. There were no significant differences among the PCD, PNCD and HCs in terms of age, sex, education level. Besides, the middle ear function was normal among three groups, as they all showed a type‐A tympanometry curve. The average hearing thresholds of both ears among the three groups are presented in Figure 2. PCD and PNCD patients exhibited significantly higher average PTA than HCs (*p* < 0.001, 500–8000 Hz). For cognitive assessment, 30 patients with presbycusis performed significantly poorer in MoCA scores than other groups (*p* < 0.001).

**TABLE 1:** Demographics among presbycusis patients and HCs.

	PCD (*n* = 30)	PNCD (*n* = 30)	HCs (*n* = 40)	*p* Value
Age (year)	63.20 ± 7.33	62.20 ± 7.05	61.55 ± 3.72	0.529^a^
Sex (M/F)	14/16	12/18	14/26	0.776^b^
Education (years)	10.47 ± 1.94	11.46 ± 1.71	10.70 ± 1.78	0.082^a^
PTA of left ear (dB HL)	32.33 ± 4.93	32.81 ± 5.79	16.71 ± 4.35	<0.001* ^,^ ^a^
PTA of right ear (dB HL)	32.74 ± 7.28	32.86 ± 6.08	16.46 ± 4.20	<0.001* ^,^ ^a^
Average PTA of both ears (dB HL)	32.54 ± 4.78	32.83 ± 5.21	16.58 ± 3.80	<0.001* ^,^ ^a^
MoCA scores	24.53 ± 0.78	26.62 ± 0.71	26.75 ± 1.30	<0.001* ^,^ ^a^

*Note*: Data are represented as Mean ± SD.

Abbreviations: F, female; HCs, healthy controls; M, male; MoCA, Montreal Cognitive Assessment; PCD, presbycusis with cognitive decline; PNCD, presbycusis with no cognitive decline; PTA, puretone audiometry.

^a^ The *p* values are obtained by using one‐way analysis of variance.

^b^ The *p* values are obtained by using *χ* ^2^ test.

* *p* < 0.001.

**FIGURE 3:**
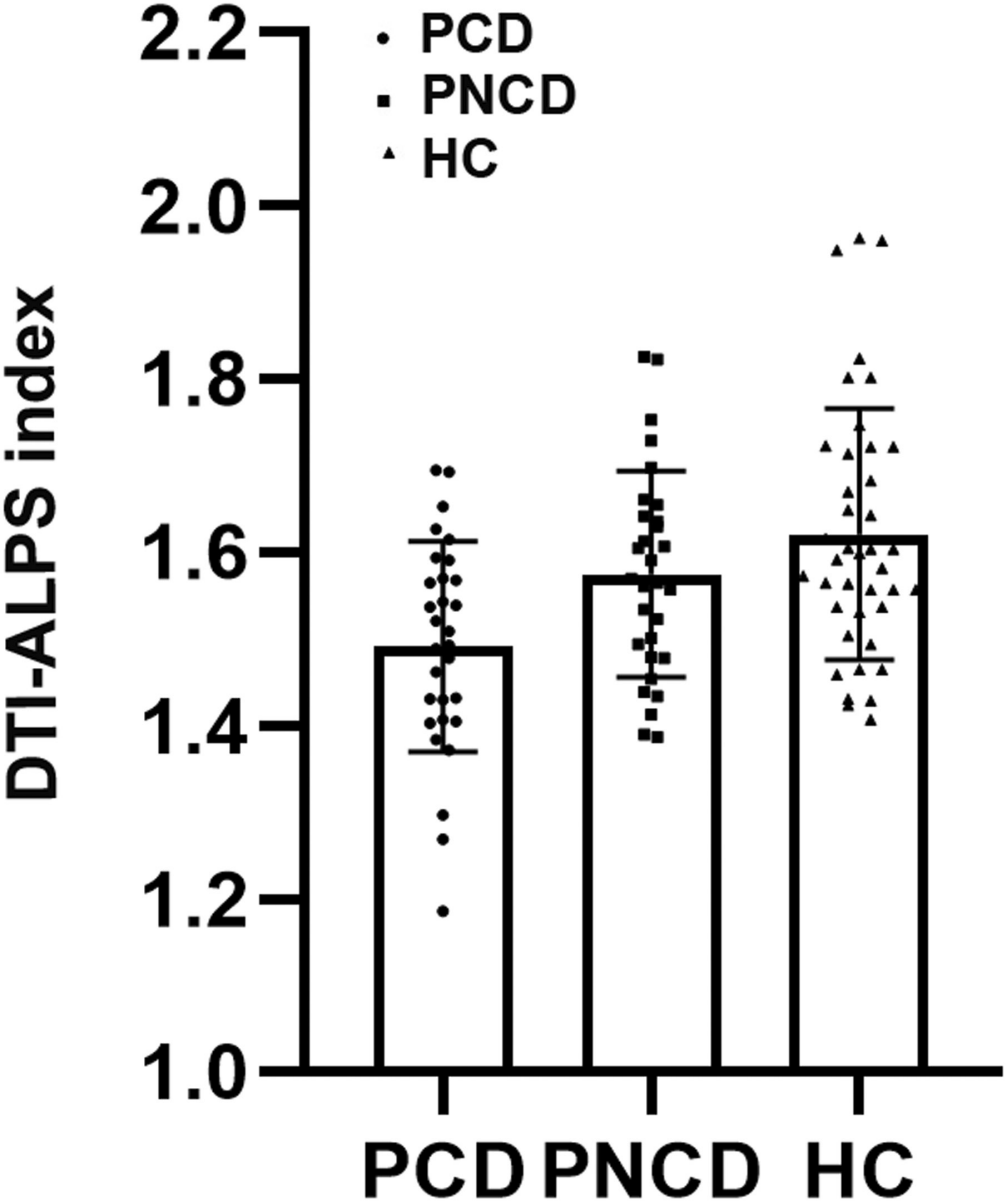
Differences between the glymphatic system functions of PCD, PNCD, and HC. The figure shows that the DTI‐ALPS index of PCD is significantly lower than that of PNCD and HC, suggesting that PCD patients have glymphatic system dysfunction. DTI‐ALPS, diffusion tensor image analysis along the perivascular space; HC, healthy controls; PCD, presbycusis patients with cognitive decline; PNCD, presbycusis patients with no cognitive decline.

The comparisons of the diffusivities data are summarized in Table 2. Using ANOVA, it showed there were significant differences in Dzzassoc and DTI‐ALPS index among the three groups. Post‐hoc analysis indicated that the DTI‐ALPS index was significantly lower in PCD patients compared to PNCD and HCs (Figure 3). Besides, Dzzassoc was significantly higher in PCD patients than in PNCD. However, there was no statistically significant difference in Dxxassoc, Dxxproj, Dyyassoc, Dyyproj, and Dzzproj among the PCD, PNCD, and HCs groups.

**TABLE 2:** Comparison of the diffusivities among presbycusis patients and HCs.

Diffusivity	PCD (*n* = 30)	PNCD (*n* = 30)	HCs (*n* = 40)	*p* Value
Dxxproj	0.00060 ± 0.00004	0.00058 ± 0.00005	0.00060 ± 0.00004	0.324
Dxxassoc	0.00063 ± 0.00007	0.00061 ± 0.00007	0.00063 ± 0.00008	0.507
Dyyproj	0.00043 ± 0.00007	0.00041 ± 0.00005	0.00039 ± 0.00006	0.084
Dyyassoc	0.00111 ± 0.00008	0.00108 ± 0.00006	0.000112 ± 0.00006	0.132
Dzzproj	0.00112 ± 0.00007	0.00109 ± 0.00007	0.00110 ± 0.00006	0.337
Dzzassoc	0.00040 ± 0.00007	0.00035 ± 0.00006	0.00037 ± 0.00006	0.010*
ALPS index	1.49147 ± 0.12153	1.57441 ± 0.11872	1.62020 ± 0.14485	<0.001**

*Note*: Data are represented as Mean ± SD. *p < 0.05, **p < 0.001.

Abbreviations: ALPS, analysis along the perivascular space; Dxxassoc, diffusivity along the x‐axis in the association fiber; Dxxproj, diffusivity along the x‐axis in the projection fiber; Dyyassoc, diffusivity along the y‐axis in the association fiber; Dyyproj, diffusivity along the y‐axis in the projection fiber; Dzzassoc, diffusivity along the z‐axis in the association fiber; Dzzproj, diffusivity along the z‐axis in the projection fiber; HCs, healthy controls; PCD, presbycusis with cognitive decline; PNCD, presbycusis with no cognitive decline.

**FIGURE 4:**
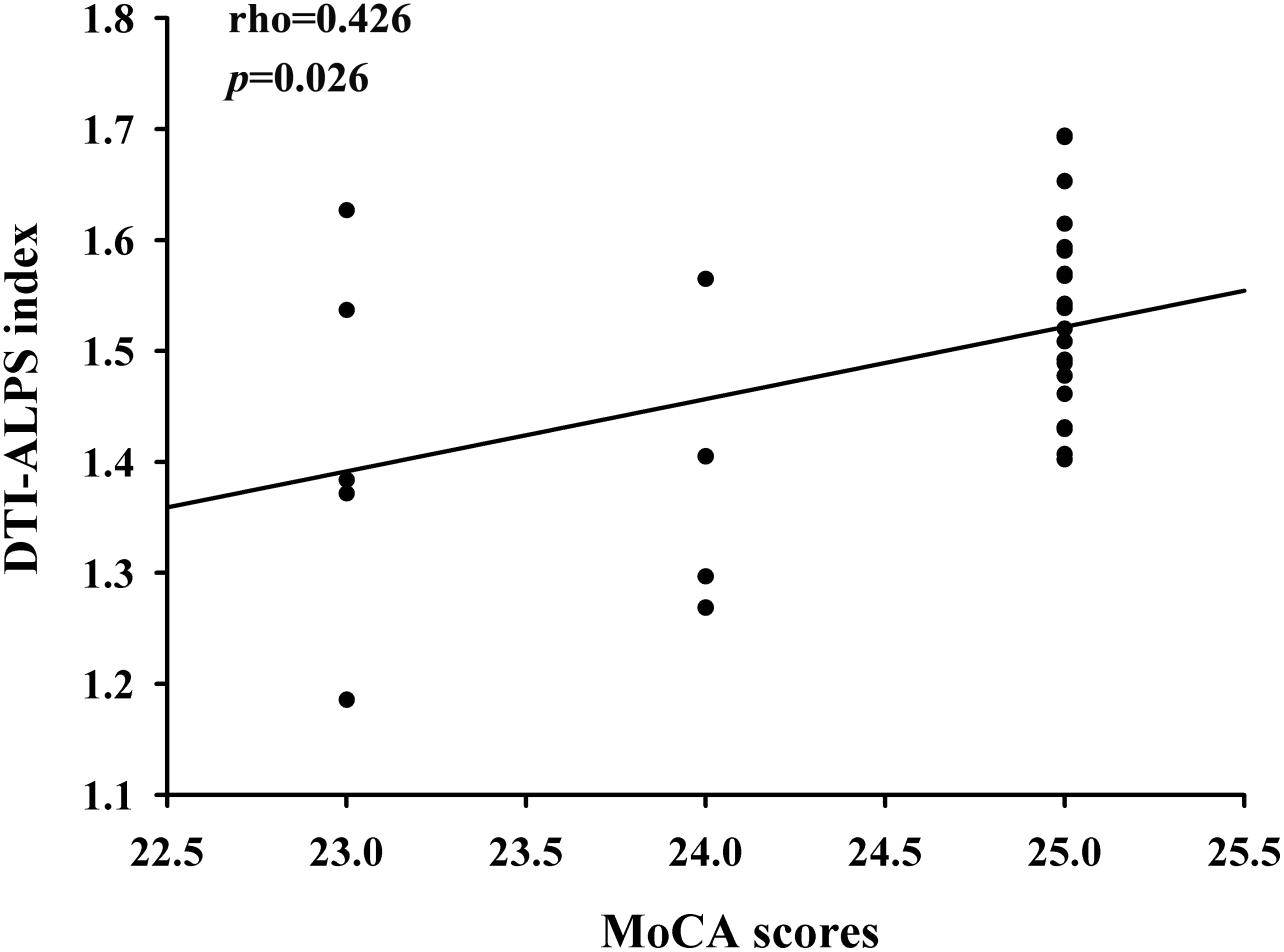
Positive correlations between diffusion tensor image analysis along the perivascular space (DTI‐ALPS) index and MoCA scores (*r* = 0.511, *p* = 0.006).

After correcting for age, gender, and education, there was a marked positive correlation between DTI‐ALPS index and MoCA scores (rho = 0.426, *p* = 0.026) (Figure 4). However, none of the other diffusivities were correlated with the clinical characteristics or MoCA scores.

## 4 DISCUSSION

This study is the first to explore the glymphatic system activity in presbycusis patients using the DTI‐ALPS method. The major finding was that presbycusis patients with cognitive impairment had glymphatic system dysfunction compared to patients without cognitive impairment and HCs. In addition, glymphatic system dysfunction correlated well with the MoCA scores in presbycusis patients. The DTI‐ALPS index may provide useful disease progression or treatment biomarkers for presbycusis patients as an indicator of modulation of glymphatic function.

Ample evidence from epidemiological studies has strongly indicated that presbycusis is independently associated with cognitive impairment, accounting for approximately 9.1% of dementia cases worldwide in middle age.^26^ Its main manifestations include worse executive function, language memory, situational and semantic long‐term memory and psychomotor processing. Therefore, presbycusis may have significant adverse effects on cognitive function. It is well known that the pathogenesis of AD is the aggregation and deposition of soluble amyloid beta (Aβ) protein in the brain. Furthermore, several recent studies have indicated that worsening hearing was associated with higher Aβ protein in age‐related hearing loss, which was measured in vivo with PET scans.^27^, ^28^, ^29^ The overall function of the glymphatic system is the clearance of the brain parenchyma from metabolic leftovers and interstitial solutes, including Aβ protein.^8^ Thus, it is reasonable to hypothesize that patients with presbycusis may have glymphatic system dysfunction, especially for those patients with cognitive decline. Moreover, glymphatic system damage caused by aging may lead to cognitive decline and neurodegenerative diseases.^30^ However, we found no significant correlation between age and glymphatic system dysfunction in this study. Whether and how aging plays a crucial role in the glymphatic system function of presbycusis still remains to be further investigated.

The DTI‐ALPS method can be used to evaluate the diffusivity along the direction of the perivascular space compared with the direction of projection fibers and association fibers on a slice at the level of the lateral ventricle body.^17^ It is deducible that the DTI‐ALPS index was associated with cognitive function. Although this method is not well established for assessing glymphatic system function in humans, it has the advantage of not requiring intrathecal administration of tracers or gadolinium‐based contrast enhancements. Previous studies have used the DTI‐ALPS method to assess glymphatic system function and demonstrated glymphatic system dysfunction in several neurological diseases with cognitive impairment. Taoka et al. firstly observed a significant positive correlation between the DTI‐ALPS index and mini‐mental status exam (MMSE) scores in AD patients.^17^ Liang et al. demonstrated impairment of the glymphatic system in dementia patients by decreased DTI‐ALPS index, including the AD, mild cognitive impairment (MCI), vascular cognitive impairment (VCI).^31^ Recently, Hsu et al. found that ALPS index is a significant mediator in the relationship between the deposition of amyloid and tau proteins and cognitive impairment, which may indicate that glymphatic dysfunction contributes to the pathogenesis of AD.^32^ Chang et al. confirmed that the association of the ALPS index and cognition was fully mediated by gray matter reserve in the amygdala, thalamus, and hippocampus in patients with young‐onset AD.^33^ Our results of the current study showed a significant positive correlation between the diffusivity along the perivascular space and the MoCA score, indicating impaired water diffusivity in the direction of the perivascular space in relation to the severity of presbycusis in areas with projection or association fiber dominance.^7^ According to these above findings, we suggest that the DTI‐ALPS method is useful for investigating glymphatic system function and correlates with cognitive impairment in neurological disorders.

Several main limitations of the current study should be acknowledged. First, this study was cross‐sectional with a relatively small sample size. Therefore, it is difficult to make a direct causal inference about the relationship between changes in glymphatic system function and presbycusis patients with/without cognitive decline. Further longitudinal studies and larger samples are required to confirm the current findings. Second, although the DTI‐ALPS index measured in the periventricular area may represent the integrity of the focal lymphatic system, an overall assessment of the focal perivascular spread was impossible because ALPS was designed to specifically measure the DTI index only in the periventricular area. Moreover, the ROI was manually placed, which may be a subjective factor in our measurement. Finally, although earplugs have been used to minimize the noise generated by MRI scans, we were unable to eliminate the possibility that noise might affect the results. This should be taken into consideration in future research.

## 5 CONCLUSIONS

Our results using the DTI‐ALPS method suggested a remarkable decrease in glymphatic activity in presbycusis patients with cognitive impairment, and positive correlation between the diffusivity along the perivascular space and MoCA scores was demonstrated. The evaluation of DTI‐ALPS demonstrates impairment of the glymphatic system in presbycusis patients and may explain the effects of age‐related hearing loss on increased risk of developing dementia.

## CONFLICT OF INTEREST STATEMENT

The authors declare that there is no potential conflict of interests regarding the publication of this paper.

## Data Availability

The data that support the findings of this study are available from the corresponding author upon reasonable request.
